# Interaction of the amyloid precursor protein-like protein 1 (APLP1) E2 domain with heparan sulfate involves two distinct binding modes

**DOI:** 10.1107/S1399004714027114

**Published:** 2015-02-26

**Authors:** Sven O. Dahms, Magnus C. Mayer, Dirk Roeser, Gerd Multhaup, Manuel E. Than

**Affiliations:** a Protein Crystallography Group, Leibniz Institute for Age Research (FLI), Beutenbergstrasse 11, 07745 Jena, Germany; b Institute of Chemistry and Biochemistry, Freie Universität Berlin, Thielallee 63, 14195 Berlin, Germany; c Miltenyi Biotec GmbH, Robert-Koch-Strasse 1, 17166 Teterow, Germany; d Department of Pharmacology and Therapeutics, McGill University Montreal, Montreal, Quebec H3G 1Y6, Canada

**Keywords:** APP-like protein 1, heparan sulfate binding domain, protein heparin complex, Alzheimer’s disease

## Abstract

Beyond the pathology of Alzheimer’s disease, the members of the amyloid precursor protein (APP) family are essential for neuronal development and cell homeostasis in mammals. APP and its paralogues APP-like protein 1 (APLP1) and APP-like protein 2 (APLP2) contain the highly conserved heparan sulfate (HS) binding domain E2, which effects various (patho)physiological functions. Here, two crystal structures of the E2 domain of APLP1 are presented in the apo form and in complex with a heparin dodecasaccharide at 2.5 Å resolution. The apo structure of APLP1 E2 revealed an unfolded and hence flexible N-terminal helix αA. The (APLP1 E2)_2_–(heparin)_2_ complex structure revealed two distinct binding modes, with APLP1 E2 explicitly recognizing the heparin terminus but also interacting with a continuous heparin chain. The latter only requires a certain register of the sugar moieties that fits to a positively charged surface patch and contributes to the general heparin-binding capability of APP-family proteins. Terminal binding of APLP1 E2 to heparin specifically involves a structure of the nonreducing end that is very similar to heparanase-processed HS chains. These data reveal a conserved mechanism for the binding of APP-family proteins to HS and imply a specific regulatory role of HS modifications in the biology of APP and APP-like proteins.

## Abstract 2

Two X-ray structures of APLP1 E2 with and without a heparin dodecasaccharide are presented, revealing two distinct binding modes of the protein to heparan sulfate. The data provide a mechanistic explanation of how APP-like proteins bind to heparan sulfates and how they specifically recognize nonreducing structures of heparan sulfates.

## 1. Introduction   

The family of amyloid precursor protein (APP)-like proteins (APLP1 and APLP2) has been the focus of intensive research in recent decades owing to the involvement of APP and its cleavage product Aβ in the pathology of Alzheimer’s disease (Dawkins & Small, 2014 ▶; Muller & Zheng, 2012 ▶; Blennow *et al.*, 2006 ▶). Although all APP-family members undergo regulated intramembrane proteolysis, amyloidogenic peptides are exclusively released upon the cleavage of APP (Jacobsen & Iverfeldt, 2009 ▶; Lichtenthaler *et al.*, 2011 ▶). However, the physiological functions of this protein family are not well understood. Knockout studies with mice impressively demonstrated (i) perinatal lethality if all APP-family proteins are deleted (Herms *et al.*, 2004 ▶) and (ii) functional overlap if different combinations of APP family proteins are present (Heber *et al.*, 2000 ▶; Anliker & Muller, 2006 ▶). This observation is supported by the conserved domain architecture of these type I transmembrane proteins, which has been studied in detail for APP (Coburger *et al.*, 2014 ▶). The ectodomains of all APP-family proteins share the N-terminal E1 domain (Dahms *et al.*, 2010 ▶) and the central E2 domain (Dulubova *et al.*, 2004 ▶; Wang & Ha, 2004 ▶; Dahms *et al.*, 2012 ▶). Two intrinsically unstructured and flexible regions, called the acidic region (AcD) and the juxtamembrane region (JMR), connect these two domains and tether the whole ectodomain to the transmembrane helix (Coburger *et al.*, 2013 ▶). Consequently, the complex domain structure found for APP-family proteins suggests a multitude of physiological functions (Coburger *et al.*, 2014 ▶).

Recent studies have supported a ligand-like role of the soluble APP ectodomain (Reinhard *et al.*, 2013 ▶) that involves binding of the E2 domain to heparan sulfate (HS) proteoglycans (HSPGs), namely glypicans and syndecans. Structural studies of the E2 domains of APP (Wang & Ha, 2004 ▶; Dahms *et al.*, 2012 ▶), APLP1 (Lee *et al.*, 2011 ▶; Xue *et al.*, 2011 ▶) and the homologous APP-like protein APL-1 (Hoopes *et al.*, 2010 ▶) from *Caenorhabditis elegans* revealed high overall similarity of the structures. For APP E2 a metal-binding site was identified that modulates the conformational flexibility of the protein (Dahms *et al.*, 2012 ▶). In addition, zinc ions influenced the oligomerization state of APLP1 E2 and induced the clustering of APLP1 at the cell surface (Mayer *et al.*, 2014 ▶). Binding of heparin to APLP1 E2 has been demonstrated biochemically (Lee *et al.*, 2011 ▶) and by soaking of crystals with a heparin hexasaccharide (Xue *et al.*, 2011 ▶). Metal and heparin binding have been shown to be conserved among APP-family proteins (Xue *et al.*, 2011 ▶; Dahms *et al.*, 2012 ▶) and might be functionally related (Multhaup *et al.*, 1994 ▶). These functionalities could also influence the binding of E2 to inter­action partners, for example F-spondin (Ho & Südhof, 2004 ▶; Hoe *et al.*, 2005 ▶) and SorlA (Willnow & Andersen, 2013 ▶). The proposed function of glypicans and syndecans as co-receptors for APP imply a specific interaction of E2 and these HSPGs (Reinhard *et al.*, 2013 ▶).

HS chains are post-translationally attached to the reducing end of a tetrasaccharide linker to a serine residue of the core protein (Häcker *et al.*, 2005 ▶). The sugar chain is synthesized as a polymer of disaccharide units containing glucuronic acid (GlcA) and *N*-acetylglycosamine (GlcNAc). Modifications of HS include the epimerization of GlcA to iduronic acid (IdoA) and the sulfation of IdoA as well as GlcNAc. Successive sulfation of HS chains results in the formation of sulfate-rich regions (NS-domains) that include specific sulfation patterns. Consequently, HS chains are highly variable and contain specific motifs that increase the affinity for glycosamino­glycan-binding proteins (Kreuger *et al.*, 2006 ▶), as demonstrated, for example, in thrombin–anti­thrombin (Li *et al.*, 2004 ▶) and integrin–VLA-4 (Schlesinger *et al.*, 2009 ▶) complexes. The modification of HS chains by heparanase can regulate the activity of HSPGs (Fux *et al.*, 2009 ▶), for example the binding of syndecan 1 to the mitogen lacritin (Ma *et al.*, 2006 ▶). A similar mechanism might influence the interaction of APP family proteins and HSPGs.

We have crystallized APLP1 E2 with and without a heparin dodecasaccharide. Comparison of the different E2 structures confirmed the remarkable conformational flexibility of this subdomain. Our data provide a structural basis for specific heparan sulfate recognition at the nonreducing end by the conserved E2 domain of APP-family proteins.

## 2. Materials and methods   

### 2.1. Expression and purification   

The APLP1 E2 domain was expressed and purified as described previously (Mayer *et al.*, 2014 ▶). Briefly, expression was performed in *Pichia pastoris* with the pPICZα vector system (Invitrogen). The expression construct comprised amino acids 290–495 of human APLP1 E2. *P. pastoris* was precultured in BMGY, pelleted and resuspended in BMMY expression medium with 0.5% methanol at an *A*_600_ of 1.0. The supernatant was collected 24 h after methanol induction and was initially purified using a HiTrap Blue HP (GE Healthcare) column. Since we observed mixed expression of *N*-glycosylated and nonglycosylated APLP1 E2 with a low content of glycosylated protein, we removed the latter by lectin affinity chromatography (Concanavalin A Sepharose, Sigma) before gel filtration on a HiLoad 16/60 Superdex 200 column (GE Healthcare) to obtain a homogenous protein solution.

### 2.2. Crystallization and structure determination   

Native APLP1 E2 (10 mg ml^−1^ in 5 m*M* Tris–HCl pH 6.8, 300 m*M* NaCl) crystals without heparin were grown in sitting drops at 20°C by mixing equal volumes of protein solution and reservoir solution consisting of 0.1 *M* sodium citrate pH 5.6, 0.9 *M* (NH_4_)H_2_PO_4_, 25% glycerol. For co-crystallization setups, protein solution (100 m*M* NaCl, 5 m*M* Tris–HCl pH 8.0) and heparin dodecasaccharide (dp12; Dextra Laboratories) were mixed in an equimolar ratio with a final concentration of 0.5 m*M*. Diffraction-quality crystals were grown by the sitting-drop vapour-diffusion method at 20°C by mixing equal volumes of protein–heparin complex solution and reservoir solution consisting of 0.1 m*M* MES–NaOH pH 6.1–6.3, 8–11% PEG 20 000. For cryoprotection, crystals were incubated stepwise for 10 min in reservoir solution supplemented with 12.5% and finally 25% glycerol.

### 2.3. Data collection   

Diffraction data were collected at 100 K on BESSY II beamline 14.1 at the Helmholtz-Zentrum Berlin (HZB; Mueller *et al.*, 2012 ▶) and were processed with *XDS* (Kabsch, 2010 ▶) and programs from the *CCP*4 suite (Winn *et al.*, 2011 ▶). Data-collection statistics are given in Table 1 ▶. To verify that a putative zinc ion was involved in crystal contacts in APLP1 E2–heparin crystals (see below) X-ray fluorescence scans were performed (Supplementary Fig. S1) and complete anomalous data sets were collected at either end of the *K* absorption edge (Table 1 ▶).

### 2.4. Structure determination, refinement and analysis   

Initial attempts to use the available structure of APLP1 E2 (PDB entry 3pmr; Lee *et al.*, 2011 ▶) as a search model for molecular replacement (MR) of apo APLP1 crystals failed, implying the presence of major conformational differences. Finally, the search model was truncated stepwise at the N- and C-temini and MR was performed using *MOLREP* (Vagin & Teplyakov, 2010 ▶) with a locked rotation function. Two APLP1 E2 molecules were identified in the asymmetric unit. The structure was refined to 2.6 Å resolution using *CNS* v.1.3 (Brunger, 2007 ▶) and *PHENIX* (Adams *et al.*, 2010 ▶). Refinement statistics are given in Table 1 ▶. In *PHENIX* restrained individual *B*-factor refinement was performed in combination with TLS refinement, selecting the individual protein chains as TLS groups. Initially, noncrystallographic (NCS) restraints were applied during refinement. However, NCS restraints prevented decrease of the *R* factors in later refinement steps and were removed. All main-chain angles fall into the most favoured and additionally allowed regions of the Ramachandran plot (Ramachandran & Sasisekharan, 1968 ▶).

The phase problem of the APLP1 E2–heparin structure was solved by a combination of MR and SAD. MR was performed in *Phaser* (McCoy *et al.*, 2007 ▶) using the available structure of APLP1 E2 (PDB entry 3pmr; Lee *et al.*, 2011 ▶). Two APLP1 E2 molecules were identified in the asymmetric unit, which is characterized by an exceptionally high solvent content of ∼77% and very large solvent channels of >100 Å in diameter. The initial electron density was very weak around the loop regions connecting the helical segments of APLP1 E2. Phase information from MR was used to locate a zinc ion involved in crystallographic interactions. Using the zinc peak data set, SAD phasing and density modification were performed in *SHARP* (de La Fortelle & Bricogne, 1997 ▶; Table 1 ▶). The model bias-free experimental electron-density map was used to verify and extend the initial model.

A strong electron-density peak indicated the presence of a metal ion connecting two symmetry-related APLP1 E2 molecules in the heparin complex crystals. The identity was clarified by X-ray fluorescence measurements (Supplementary Fig. S1) and the calculation of an element-specific ‘difference DANO map’ (Supplementary Fig. S2). The tetrahedral coordination sphere of the zinc ion is formed by a bidentate zinc chelator in addition to the histidine side chains (His415 and His548) of the APLP1 E2 symmetry mates (Supplementary Fig. S2). The exact identity and source of this zinc chelator remained unclear. For structure refinement, the electron density of the compound was approximated by fitting single O atoms into the electron density (Supplementary Fig. S2).

Finally, the structure was refined to 2.5 Å resolution using *CNS* v.1.3 (Brunger, 2007 ▶) and *PHENIX* (Adams *et al.*, 2010 ▶). Refinement statistics are given in Table 1 ▶. Consecutive runs of grouped occupancy and *B*-factor refinement of the individual heparin chains in *CNS* resulted in average occupancies close to 1.0 (∼1.0 for heparin chain *a* and ∼0.8 for heparin chain *b*). For this reason, the occupancies of both heparin chains were set to 1.0 and only the *B* factors were refined. In *PHENIX*, restrained individual *B*-factor refinement was performed in combination with TLS refinement, selecting the individual protein chains and individual heparin chains as TLS groups. All main-chain angles of the protein backbone fall into the most favoured and additionally allowed regions of the Ramachandran plot (Ramachandran & Sasisekharan, 1968 ▶). The carbohydrates were refined using library values from *CNS* v.1.3 (Brunger, 2007 ▶). Bond lengths, dihedral angles and bond angles of substituents missing in the *CNS* library were derived from saccharide structures from the Cambridge Structural Database (CSD; Allen, 2002 ▶). The used heparin preparation contained 2.5 sulfo groups per IdoAp2S-GlcNpS6S subunit and the occupancies of the sulfate groups were modelled correspondingly with a value of 0.83. Model building was performed in *Coot* (Emsley *et al.*, 2010 ▶) and *MAIN* (Turk, 2013 ▶).

Composite annealed OMIT maps were calculated in *CNS* v.1.3 (Brunger, 2007 ▶), omitting 2.5% of the final model. *F*_o_ − *F*_c_ kicked maps were calculated in *PHENIX* (Adams *et al.*, 2010 ▶). Element-specific ‘difference DANO maps’ were calculated as described previously (Than *et al.*, 2005 ▶). *PyMOL* (http://www.pymol.org) was used for molecular graphics and sequence-based structure alignments. Surface electrostatics were calculated with *APBS* (Baker *et al.*, 2001 ▶).

The structure factors and coordinates of APLP1 E2 in complex with heparin and of the apo structure of APLP1 E2 have been deposited in the Protein Data Bank (PDB) with accession codes 4rda and 4rd9, respectively.

### 2.5. Thermal denaturation analysis   

Thermal denaturation analysis (also called a Thermofluor assay; Ericsson *et al.*, 2006 ▶) was performed in 50 m*M* NaH_2_PO_4_/Na_2_HPO_4_ pH 5.7, 100 m*M* NaCl, 15× SYPRO Orange (Life Technologies), adding 10 µ*M* APLP1 E2 and 50 µ*M* of the heparin oligosaccharides. All heparin oligosaccharides contained a Δ^4^UAp2S moiety at the nonreducing end following the chemical structure Δ^4^UAp2S-GlcNpS6S-(IdoAp2S-GlcNpS6S)_*n*_, with *n* = 0 for dp2 (Sigma–Aldrich), *n* = 1 for dp4, *n* = 3 for dp8, *n* = 5 for dp12 and *n* = 7 for dp16 (all from Dextra Laboratories). Melting curves were determined using an IQ5 real-time PCR cycler (Bio-Rad), measuring the increase in fluorescence at excitation and emission wavelengths of 490 and 575 nm, respectively. Heating was performed in 0.5°C steps with a subsequent dwell time of 15 s. The melting temperatures correspond to the inflection points of the melting curves, which were determined with the data-analysis module of the *IQ*5 software (v.2.1.97.1001; Bio-Rad). All measurements were performed in triplicate and the error bars shown represent the respective standard deviations.

## 3. Results   

### 3.1. A novel crystal form of apo APLP1 E2   

**Figure 1:**
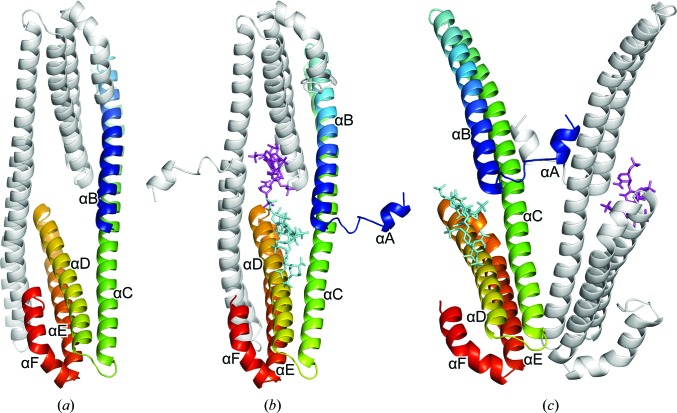
Overall structures of the APLP1 E2 domain and of the (APLP1 E2)_2_–(heparin)_2_ complex. (*a*) Heparin-free APLP1 E2 is shown in cartoon representation. Individual protomers found in the asymmetric unit are indicated in grey and in a rainbow colour scheme indicating the extension of the peptide chain from the N-­terminus (blue) to the C-terminus (red). (*b*) The (APLP1 E2)_2_–(heparin)_2_ complex showing the protein in cartoon representation and the bound saccharide as a stick model. (*c*) An alternative, parallel-oriented, dimeric assembly present in the asymmetric unit of the (APLP1 E2)_2_–(heparin)_2_ complex structure. The same colour scheme as shown in (*a*) is used to discriminate the individual protein chains of the asymmetric unit. Individual heparin chains are coloured magenta (heparin chain *a*) or cyan (heparin chain *b*).

We identified a new orthorhombic crystal form of the E2 domain of APLP1. The respective structure was built and refined to 2.6 Å resolution (Table 1 ▶). N-terminal and C-terminal truncations of the search model (PDB entry 3pmr; Lee *et al.*, 2011 ▶) were required to solve the structure by molecular replacement, implying major conformational differences. Indeed, the N-terminal helix αA was not observed in the new crystal form (although it was present in the protein construct used), suggesting that it is rather flexible and becomes sufficiently stabilized only by certain intermolecular interactions. The final structure is composed of five α-helices (Fig. 1 ▶ *a*). Comparable to the E2 structures of other APP-family proteins (Wang & Ha, 2004 ▶; Hoopes *et al.*, 2010 ▶; Dahms *et al.*, 2012 ▶), a coiled-coil-like fold is formed by helix αB (Gly302–Gln338) and the first half of helix αC (Lys344–Arg379). The C-terminal parts of helices αC (Val380–Leu399), αD (Ala406–Val437), αE (Pro439–Asp468) and αF (Pro471–Gly491) constitute a four-helix bundle. Two APLP1 E2 protomers are arranged in an antiparallel, dimeric assembly within the asymmetric unit of the new crystal form (Fig. 1 ▶ *a*). A similar arrangement has been described for APLP1 E2 in previous studies and was functionally connected to heparin binding (Xue *et al.*, 2011 ▶). Despite the similar overall topologies of these dimeric APLP1 E2 assemblies, the C^α^ r.m.s.d. value of approximately 1.5 Å (for helices αB7–αF) is rather large between the crystal structure described here and that solved previously (Lee *et al.*, 2011 ▶).

### 3.2. Co-crystals of APLP1 E2 and heparin dodecasaccharide: overall structure   

In previous studies, apo APLP1 crystals were used to investigate the interaction with heparin hexasaccharides by soaking experiments (Xue *et al.*, 2011 ▶). Although binding of heparin was observed by this approach, parts of the ligand were unordered and several important details of the binding mode of elongated heparin molecules remained unresolved. To clarify these issues, we decided to co-crystallize APLP1 E2 in the presence of a heparin dodecasaccharide. Prior to crystallization, the protein–heparin complex was prepared by mixing the protein and saccharide in an equimolar ratio. The heparin–protein complex crystallized in space group *P*3_1_21 with a very high solvent content of 77% (Table 1 ▶). The structure was solved by a combination of molecular replacement and single anomalous dispersion, employing anomalous scattering from a bound Zn^2+^ ion. Refinement was performed to a resolution of 2.5 Å and resulted in good stereochemistry (Table 1 ▶). Interestingly, we identified a zinc ion in the APLP1 E2–heparin complex crystals that was coordinated by two symmetry-related APLP1 E2 molecules and a bidentate chelating compound, although no Zn^2+^-containing reagent was purposely added during crystallization (see §[Sec sec2]2 for details and Supplementary Figs. S1 and S2). Also, none of the previously described histidine-rich metal-binding sites (Dahms *et al.*, 2012 ▶; Mayer *et al.*, 2014 ▶) were found to be occupied by zinc in the crystals described here or in purposely Zn^2+^-soaked crystals (data not shown).

Two APLP1 E2 molecules associated with two different heparin chains were observed in the asymmetric unit (Fig. 1 ▶ *b*). In contrast to the apo form, the short N-terminal α-helix αA (Asp292–Phe298) is well defined in the electron-density map in this trigonal crystal form of the complex. The protein forms an antiparallel dimeric arrangement in the asymmetric unit as observed for the heparin-free APLP1 E2 structure (see above). Evaluation of all of the crystallographic interactions, however, revealed an alternative definition of the asymmetric unit, now representing a parallel protein dimer (Fig. 1 ▶ *c*). This alternative arrangement is largely stabilized by the N-termini of APLP1 E2 (Asp292–Ile304). Analysis with the *PISA* server (Krissinel & Henrick, 2007 ▶) classified both assemblies as stable, with buried surface areas of ∼1860 Å^2^ (antiparallel) and ∼1520 Å^2^ (parallel). A similar parallel crystallographic dimer contact has previously been reported in an ortho­rhombic crystal form (Lee *et al.*, 2011 ▶). The unusual crossed coiled coil formed by the N-terminus of this assembly was considered to be a crystallization artifact (Lee *et al.*, 2011 ▶). We conclude, however, that both contacts can be formed in the crystalline state and that both contacts are of similar energetic importance for the stabilization of the crystal and the asymmetric unit. A careful sequence-conservation analysis also revealed a similar degree of evolutionary conservation for both interaction interfaces (Supplementary Figs. S3*a* and S3*b*). According to these data, none of the dimeric assemblies preferentially correlates with an outstanding functional role based on evolutionary constraints. A final conclusion on the dimer and the dimeric contacts formed by APLP1 E2 can thus only be drawn from further in-depth studies.

**Figure 2:**
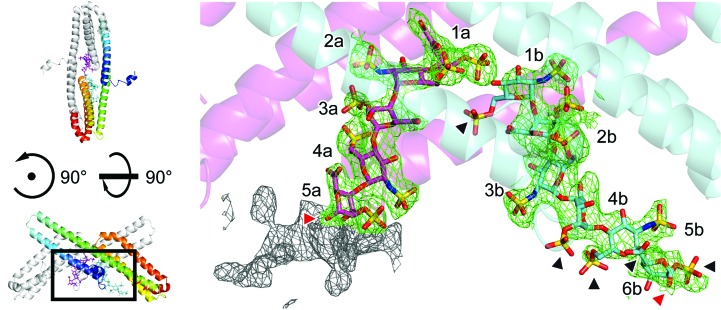
Heparin molecules bound in the (APLP1 E2)_2_–(heparin)_2_ complex structure. The orientation corresponds to Fig. 1 ▶(*b*) rotated by 90° around the *z* and *x* axes (left). The heparin-binding sites (right) are shown perpendicular to the twofold noncrystallographic symmetry axis of the antiparallel (APLP1 E2)_2_ assembly. APLP1 E2 is shown as transparent cartoon representation and individual protein chains are coloured magenta (chain *a*) and cyan (chain *b*). The C atoms of individual heparin chains are coloured likewise. The heparin chains are consecutively numbered, starting with the nonreducing end positioned in the centre of the APLP1 E2 assembly. The 2*F*_o_ − *F*_c_ simulated-annealing composite OMIT electron-density map is contoured at 1σ (green and grey mesh, respectively). The grey parts of the 2*F*_o_ − *F*_c_ simulated-annealing composite OMIT electron-density map highlight a region at the reducing end of heparin chain *a* that was ambiguous and hence was not included in the model. The reducing ends of both heparin chains (C1 positions) are marked with red arrowheads. Black arrowheads mark substituents of sugar residues that were modelled with an occupancy of 0 owing to weak electron density.

Two heparin chains with seven (chain *a*) and six (chain *b*) sugar residues were identified in the asymmetric unit of our protein–heparin complex crystals (Figs. 1 ▶ *b* and 1 ▶ *c*). The electron-density map clearly indicated the placement of the distinct sugar chains with the nonreducing ends oriented towards the centre of the parallel APLP1 E2 dimer (Fig. 2 ▶). Correspondingly, the reducing end of heparin is directed towards the huge solvent channels of greater than 100 Å in diameter. At the reducing end of the heparin chain the electron density for 2–3 sugar residues beyond residue 5a was less well defined (Fig. 2 ▶; grey-coloured electron density). The exact location of these sugar rings was ambiguous. Attempts to extend the model to this region always resulted in an irregular geometry of the heparin chain. We thus conclude that this part is characterized by increased conformational flexibility, as also indicated by the increased crystallographic *B* factors of residue 5a (see below).

The heparin preparation used consisted of alternating 2-*O*-sulfo-α-l-idopyranosyluronic acid (IdoAp2S) and 2-deoxy, 2-sulfamido, 6-*O*-sulfo-α-d-glucopyranose (GlcNpS6S) moieties, exposing 4-deoxy-α-l-*threo*-2-sulfo-hex-4-enopyranosyluronic acid (Δ^4^UAp2S) at the nonreducing end. All GlcNpS6S and IdoAp2S residues were built in the ^4^ *C*_1_ and ^1^ *C*_4_ chair conformations, respectively. The sugar rings and the sulfate substituents of the heparin chains resulted in prominent features in the electron density (Fig. 2 ▶). In particular, the presence of two sulfate groups and their typical distances to the ring C atoms unambiguously identified GlcNpS6S, *e.g.* in positions 2a, 4a and 3b. In comparison IdoAp2S contains only one sulfate substituent at the ring carbon C2 and a characteristic carboxyl group at C5 (*e.g.* in positions 3a, 5a and 2b), clearly defining the registry of the alternating sugar rings. At both ends of heparin chain *b*, however, the electron density is less well defined, indicating increased flexibility of heparin (Fig. 2 ▶). In particular, the sulfate substituents of sugar residues without any specific protein contacts are flexible (see below).

### 3.3. Specific recognition of heparin chain ends by APLP1 E2   

**Figure 3:**
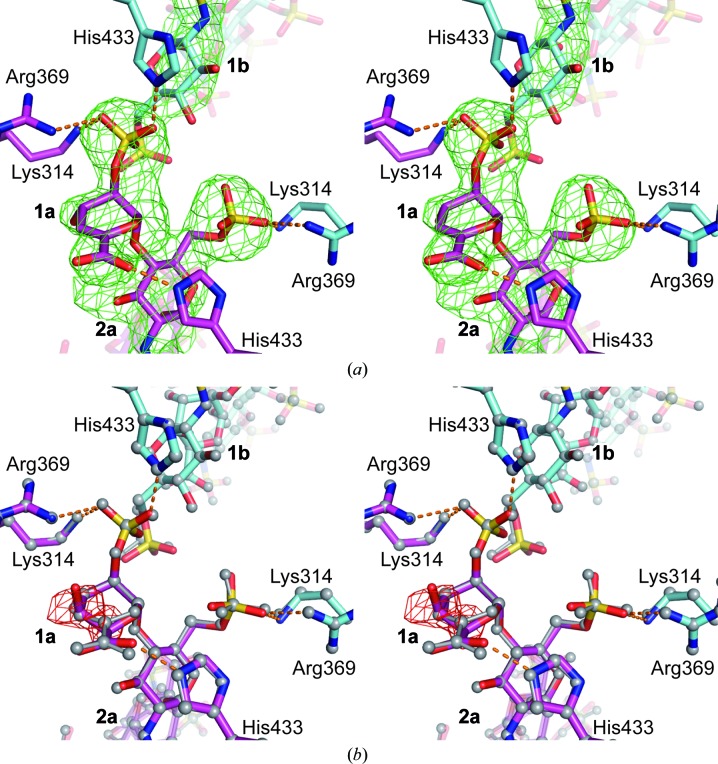
Close-up view of the nonreducing end of heparin chain *a* with Δ^4^UAp2S. The stereo panels show a close-up view of the centre of the antiparallel (APLP1 E2)_2_ assembly. Selected amino-acid side chains involved in heparin binding are shown as stick models and are coloured according to the individual protein chains in magenta (chain *a*) or in cyan (chain *b*). C atoms of individual heparin chains are coloured likewise. Specific interactions between amino-acid side chains and sugar residues are highlighted with orange dashes. Amino-acid side chains and sugar residues are numbered. (*a*) The *F*_o_ − *F*_c_ kicked heparin OMIT electron-density map is contoured at 3.5σ (green mesh). (*b*) Substitution of Δ^4^UAp2S by IdoAp2S at the nonreducing end of heparin chain *a*. Colours are as in (*a*). The final structure with Δ^4^UAp2S at the nonreducing end [as shown in (*a*)] is shown for comparison as a grey-coloured ball-and-stick model. The *F*_o_ − *F*_c_ kicked heparin OMIT electron-density map is contoured at −4.0σ (red mesh).

We observed major differences between the binding modes of the two heparin chains to APLP1 E2. Our co-crystal structure revealed unique features of an asymmetric interaction of the nonreducing end of heparin chain *a* with the protein. The electron density at the centre of the antiparallel APLP1 assembly clearly indicated the presence of a dehydrated IdoAp2S (Δ^4^UAp2S) at the terminus of chain *a* (Fig. 3 ▶ *a*). Such unsaturated sugar moieties at the nonreducing end result from the heparin preparation process by heparinase digestion. Owing to the eliminase reaction mechanism of the employed enzymes, a double bond is formed between C4 and C5 (Linhardt *et al.*, 1986 ▶). This reaction consequently results in loss of the hydroxyl group at the C4 position in Δ^4^UAp2S compared with IdoAp2S. Indeed, in our structure no electron density was observed for the C4 hydroxyl group, and the ^1^ *H*_2_ isomer of Δ^4^UAp2S fitted nicely into the electron-density map (Fig. 3 ▶ *a*). For comparison we replaced Δ^4^UAp2S and refined the structure with IdoAp2S at the nonreducing end (Fig. 3 ▶ *b*). In this case, however, negative peaks were observed in the electron-density map, confirming the absence of the C4 hydroxyl group as well as a different conformation of the C4 and C5 ring atoms of IdoAp2S. Refinement of IdoAp2S at the nonreducing end of heparin chain *a* also resulted in an increase of the *R* factor and *R*_free_ of ∼0.5%, further demonstrating the presence of Δ^4^UAp2S in the crystal. However, we did not observe any spatial restriction such that either Δ^4^UAp2S or IdoAp2S could bind with equal affinity to position 1a of the APLP1 E2 dimer.

The terminal Δ^4^UAp2S-GlcNpS6S disaccharide unit of heparin chain *a* is bound by the amino-acid side chains of Lys314, Arg369 and His433 of each protomer of the antiparallel APLP1 E2 assembly (Figs. 3 ▶ *a* and 4 ▶ *a*), albeit in different geometries. A total of four charged hydrogen bonds are formed to Δ^4^4UAp2S 1a, involving its carboxyl and 2-*O*-sulfo moieties. The interactions of APLP1 E2 with GlcNpS6S 2a involve its 2-*N*-sulfo and 6-*O*-sulfo groups.

**Figure 4:**
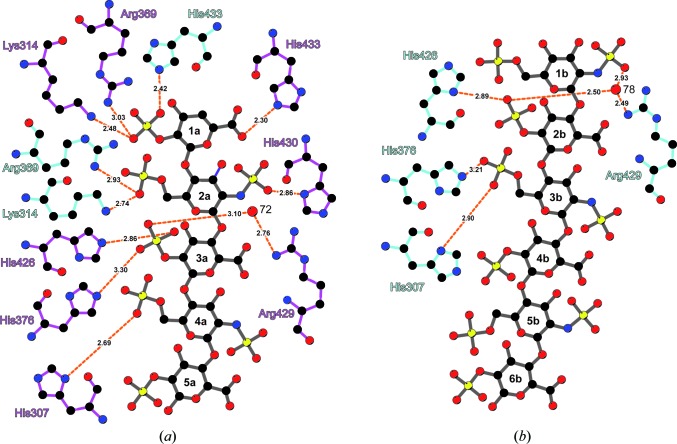
Different heparin-binding modes of APLP1 E2. The interactions of APLP1 E2 and the bound heparin chains are shown as schematic diagrams. Amino-acid side chains of individual protein chains are coloured magenta (chain *a*) or cyan (chain *b*). Heparin is coloured grey. Interactions between amino-acid side chains and sugar residues are highlighted by orange dashes with interaction distances indicated in Å. (*a*) Interactions of heparin chain *a* with APLP1 E2. (*b*) Interactions of heparin chain *b* with APLP1 E2. The interaction map was prepared with *LIGPLOT* (Wallace *et al.*, 1995 ▶).

Additional contacts to heparin chain *a* are exclusively mediated by protein chain *a* (Fig. 4 ▶ *a*); the 2-*N*-sulfo group of 2a is bound by His430, the 2-*O*-sulfo group of 3a is bound by His426 as well as by His376 and the 2-*O*-sulfo group of 4a is bound by His307. Additional water-bridged interactions are formed between 3a and Arg429. All amino-acid side chains involved in specific heparin recognition showed an exceptionally high degree of evolutionary conservation among the APP protein family (Supplementary Fig. S3*c*).

### 3.4. Binding of APLP1 E2 to continuous heparin chains   

Binding of heparin chain *b* is facilitated by only one APLP1 E2 protomer (protein chain *b*; Fig. 4 ▶ *b*). The 2-*O*-sulfo group of 2b is bound to His426 and the 6-*O*-sulfo group of 3b is bound to His307 as well as to His376. Water-bridged interactions are formed between Arg429 and the 2-*N*-sulfo and 2-*O*-sulfo groups of 1b and 2b, respectively. The lack of specific interactions results in an increase in the flexibility at the non­reducing end. As consequence, the electron density of residue 1b is weak especially for the 6-*O*-sulfate, which is not involved in any interaction (Fig. 4 ▶ *b*). Elongation of the heparin chain beyond residue 1b is hindered by the tightly bound Δ^4^4UAp2S (1a) belonging to the other heparin chain. Apparently, the sugar rings preceding 1b are highly flexible and hence invisible in the electron-density map.

**Figure 5:**
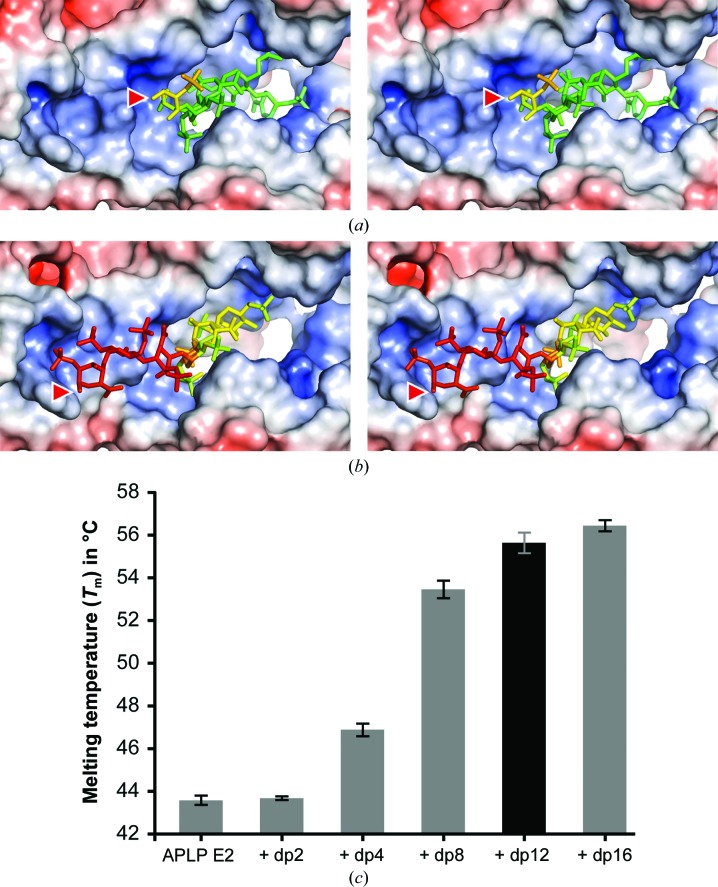
Electrostatic interactions of heparin and APLP1 E2. The stereoviews show APLP1 E2 chains *a* and *b* in the same orientation. The molecular surface is coloured according to the calculated electrostatic potential, which ranges from red (−10 *kT*/e^−^) to blue (+10 *kT*/e^−^). Heparin chains are shown as stick models coloured in a gradient according to the crystallographic *B* factor, which ranges from green (74 Å^2^) to red (179 Å^2^). Red arrowheads mark the reducing ends of heparin. (*a*) Electrostatic interactions of heparin chain *a* with APLP1 E2. (*b*) Electrostatic interactions of heparin chain *b* with APLP1 E2. (*c*) Structural stabilization of heparan sulfate by APLP1 E2. The dependence of the increase of the melting temperature of APLP1 E2 on the heparin chain length was measured by thermal denaturation assays (Thermofluor assays). The melting temperatures given correspond to the inflection points of the melting curves of APLP1 E2 without heparin and in the presence of saccharides with defined lengths of two (dp2), four (dp4), eight (dp8), 12 (dp12) and 16 (dp16) sugar rings. dp12, which was used for crystallization, is highlighted as a black bar.

The heparin chains cover positively charged surface patches of APLP1 E2 (Figs. 5 ▶ *a* and 5 ▶ *b*). Charge equalization mainly contributes to the binding of the negatively charged, extended heparin chain *b* (Fig. 5 ▶ *b*). However, specific hydrogen bonds are missing at the reducing end of this heparin chain, allowing multiple orientations of negatively charged substituents. Interestingly, the amino acids of the interaction interface of APLP1 E2 and the distant sugar moieties 4b–6b are less well conserved compared with residues involved in specific interactions (Supplementary Fig. S3*c*). The crystallographic *B* factors also indicate higher flexibility of the sugar rings 4b, 5b and 6b at the reducing end (Fig. 5 ▶ *b*). As a consequence, poor electron density was observed for several sulfate groups of these sugar residues (Fig. 2 ▶).

The charge-driven interaction of APLP1 E2 with heparin chain *b* is expected to be much weaker than the interaction with heparin chain *a* owing to the lack of the specific hydrogen-bonding network (Figs. 4 ▶ *a* and 4 ▶ *b*). This observation is supported by the different overall crystallographic *B* factors of the protein chains, although the overall structural differences are low (C^α^ r.m.s.d. ≃ 0.6 Å). For APLP1 E2 chain *a* a lower overall *B* factor of ∼60 Å^2^ was observed compared with ∼73 Å^2^ for APLP1 E2 chain *b*. Similar differences are observed for the heparin molecules, showing lower crystallo­graphic *B* factors for chain *a* (average ∼93 Å^2^; range 74–134 Å^2^; Figs. 5 ▶ *a* and 5 ▶ *b*) compared with chain *b* (average ∼145 Å^2^; range 107–179 Å^2^). Consequently, the global stabilization of APLP1 E2 by heparin chain *a* is stronger and hence indicates tighter binding. These data strongly suggest that the binding mode observed for heparin chain *a* will be preferred if specific nonreducing ends with free Δ^4^4UAp2S or IdoAp2S residues are available.

### 3.5. Structural stabilization of APLP1 E2 by heparin oligosaccharides   

The effect of heparin binding on the structural stability of APLP1 E2 was investigated by thermal denaturation assays (Fig. 5 ▶ *c*). Indeed, the addition of the dodecasaccharide used in the crystallization setups increased the melting temperature (*T*_m_) of the protein from 43.6 ± 0.2 to 55.6 ± 0.5°C. This gain of structural stability by APLP1 E2 upon heparin binding is in excellent agreement with the extensive interactions observed in the structure. The dependence of the *T*_m_ of the protein on the heparin chain length was also analysed. For this purpose, heparin chains with lengths of two to 16 sugar rings were used containing Δ^4^UAp2S at the nonreducing end. Whereas no measurable effect was observed for disaccharides, *T*_m_ was shifted to 46.9 ± 0.3°C in the presence of tetrasaccharides and to 53.5 ± 0.5°C in the presence of octasaccharides. Correspondingly, the highest gain in structural stability was reached by the binding of heparin chains with lengths of between four and eight sugar rings. These results correlate very well with the structural data, which show 5–6 well ordered sugar rings bound to APLP1 E2. Interestingly, a slight stability increase of APLP1 was induced by chain lengths beyond eight sugar rings. This observation could be explained by the occupation of a second, weaker binding site. Indeed, binding to continuous heparin chains is expected to be weaker and an increase of the average heparin chain length will increase the number of available binding sites for this binding mode.

## 4. Discussion   

We have solved the crystal structures of heparin-bound and unbound APLP1 E2 domains, each consisting of dimeric arrangements of the protomers. Beyond the differently structured N-terminal segments (N-terminal residues Asp292–Ile304), a high overall similarity of the heparin-bound and unbound APLP1 E2 structures was observed. The co-crystal structure contained an (APLP1 E2)_2_–(heparin)_2_ assembly and revealed two different heparin-binding modes of APLP1 E2.

Comparison between the structures of APLP1 E2 described in this study indicated a major conformational difference in the N-terminal part of the protein. The folding of helix αA is shown to be independent of the other structural parts of APLP1 E2. A similar degree of flexibility was observed for the N-terminal segments of the E2 domains of other APP-family proteins (Hoopes *et al.*, 2010 ▶; Lee *et al.*, 2011 ▶; Dahms *et al.*, 2012 ▶). In the APLP1 E2–heparin co-crystal structure this part adopts an α-helical fold and contributes to protein–protein interactions with symmetry-related molecules. Two different dimeric arrangements that represent the asymmetric unit of the crystal can be defined from the crystal lattice, and both assemblies are classified as stable by the *PISA* server. In contrast to other APP-like proteins, the E2 domain was shown to contribute primarily to the dimerization of APLP1 (Kaden *et al.*, 2008 ▶). Potential dimer contacts could be increased by HS binding and contribute to cell adhesion (Lee *et al.*, 2011 ▶). The formation of higher oligomer forms in the presence of HS as expected from two equally strong protein–protein contacts has not yet been reported. Consequently, the exact identity of the dimeric contacts in solution and their physiological relevance need to be clarified.

In previous studies with APP E2 we demonstrated the presence of a metal-binding site in this domain (Dahms *et al.*, 2012 ▶). Zinc binding involves the M1 site with histidine residues 382, 432 and 436, whereas copper coordination additionally involves His313 (corresponding to His307, His376, His426 and His430 in APLP1 E2). These amino acids are highly conserved among APP-family proteins, including human APLP1 and APLP2. Metal binding at M1 requires a certain conformation of helices B, C and D that is largely different from the conformation observed in heparin-bound APLP1. Thus, binding of Zn^2+^ at the M1 site would rather disfavour the binding of heparin, as observed in our crystals. A connection between heparin and zinc binding as observed for APP in previous studies (Multhaup *et al.*, 1994 ▶) could be facilitated by other mechanisms, for example increased avidity upon zinc-induced cluster formation (Mayer *et al.*, 2014 ▶). Possible opposing effects of zinc and heparin binding might differently influence, for example, protein–protein inter­actions of APLP1 E2, which needs to be evaluated by further studies. Interestingly, a dimeric crystallographic contact in the heparin-bound APLP1 E2 crystals is mediated by a bound zinc ion. In addition to one histidine side chain of two symmetry-related APLP1 protomers, an unknown chelating compound contributes to zinc coordination. Both the zinc ions as well as the zinc chelator are most likely to originate from co-purification with the polyanionic heparin. This finding indicates the presence of trace amounts of zinc in the co-crystallization setups. However, none of the previously identified Zn^2+^ ligands is part of the chelation sphere and the observed contact is probably not of physiological relevance. Rather, it is a consequence of the specific assembly of APLP1 E2 molecules in the crystal lattice.

Previous studies implied functional interplay between dimerization and heparin binding of the APLP1 E2 domain (Xue *et al.*, 2011 ▶). However, crystals soaked with a heparin hexasaccharide allow only limited conclusions about the binding of longer HS to monomers or dimers of APLP1 E2. In this study, the protein was co-crystallized with a heparin dodecasaccharide in an equimolar ratio. Consequently, the observed APLP1 E2–heparin complex is not biased by a preformed crystal lattice. Only upon co-crystallization were two different and distinct APLP1 E2–heparin interaction modes observed. The heparin-binding site is exposed to the huge solvent channels and thus is not influenced by any crystal contact, allowing the binding of extended heparin chains. The longest heparin stretch of six sugar residues is exclusively associated with one APLP1 E2 protomer (chain *b*) and extends to a surface patch distant from the centre of the dimer. No termini or sulfation patterns of the heparin chain are specifically recognized by APLP1 E2 in this mode. Only the register of the sugar moieties determines the fit to this surface patch, which thus seems to be compatible with the binding of continuous heparin chains in various sulfation states. The interaction is dominated by charge equalization between the positively charged protein surface and the heparin polyanion. A second heparin chain is tightly and specifically bound with its nonreducing terminal Δ^4^UAp2S in the centre of the APLP1 E2 dimer. It predominantly interacts with the second protein chain *a*. Both heparin chains are bound in the same orientation with regard to the protein and face each other towards the centre of the APLP1 E2 assembly. The observed binding topology thus contradicts the hypothesized inter­action of one continuous heparin chain spreading over an extended binding site of dimeric APLP1 E2 (Xue *et al.*, 2011 ▶). Our structural data rather suggest specificity of APLP1 E2 for nonreducing ends of heparin and HS exposing terminal Δ^4^UAp2S and IdoAp2S, respectively. IdoAp2S shows similar steric requirements as Δ^4^UAp2S and is expected to replace the unsaturated sugar in physiological HSPG-type interaction partners (Häcker *et al.*, 2005 ▶). A similar specificity for HS termini is expected for APP and APLP2 owing to the high sequence conservation of the interacting amino-acid side chains. This conclusion is in excellent agreement with our previous results, showing the binding of one APP E2 molecule per heparin chain (with average heparin chain lengths of between 11 and 15 sugar rings; Hoefgen *et al.*, 2014 ▶). However, heparin preparations with higher average chain lengths in addition favour less specific binding, as observed for heparin chain *b* in our structure. These conclusions are further supported by the results obtained in the thermal shift assays. The highest gain of structural stability of APLP1 E2 was observed upon the binding of heparin molecules with lengths of between four and eight sugar rings. Further increases of the heparin chain length revealed much weaker effects on the structural stability of the protein, reminiscent of less specific and hence weaker binding to continuous heparin chains. Free and protein-bound HS are modified *in vivo* by heparanases (Fux *et al.*, 2009 ▶). These enzymes cleave 1–4 links of GlcNpS6S and IdoAp2S similar to heparinase I (Linhardt *et al.*, 1986 ▶). In contrast to heparinase, cleavage of HS by heparanase results in terminal IdoAp2S moieties. Both of the resulting termini at the nonreducing ends of HS and heparin chains (saturated IdoAp2S and unsaturated Δ^4^UAp2S, respectively) fit equally well to the heparin-binding site of APLP1 E2. Interestingly, analysis of HS from kidney cells revealed that the majority of HS contains tetrasaccharides of alternating GlcA and GlcNAc in highly variable sulfation states at the nonreducing end (Wu & Lech, 2005 ▶). It has been shown that heparanase cleavage occurs specifically at the end regions of highly sulfated NS domains (Bame, 2001 ▶). The released sugar sequences contain disaccharide units of 2-*O*-sulfated IdoA as wells as 2-*O*- and/or 6-*O*-sulfated GlcN at the nonreducing end. The 2-*O*-sulfo groups of IdoAp2S 1a and IdoAp2S 3a, the 2-*N*-sulfo groups of GlcNpS6S 2a and the 6-*O*-sulfo groups of GlcNpS6S 2a and GlcNpS6S 4a are required for specific interactions in the APLP1 E2–heparin complex structure. Consequently, the observed interaction pattern strongly suggests a preference of APLP1 E2 for heparanase-modified nonreducing ends of HS. Recent studies also suggested a function of HSPGs as a co-receptor for the ectodomain of APP, showing an interaction with glypicans and syndecans (Reinhard *et al.*, 2013 ▶), which are modified by heparanase (Fransson *et al.*, 2004 ▶; Ma *et al.*, 2006 ▶; Fux *et al.*, 2009 ▶). Strikingly, this interaction was shown to be mediated by the E2 domain of APP. The conservation of the HS binding sites of the E2 domains of APP-family members implies a similar binding of APLP1 and APLP2.

**Figure 6:**
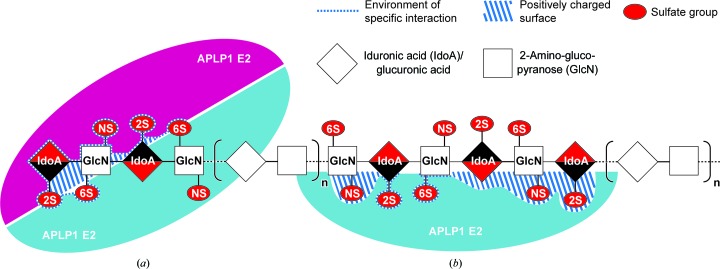
Mechanistic model of heparan sulfate binding by APLP1 E2. (*a*) Specific recognition of the highly modified nonreducing ends. (*b*) Register-dependent tethering of APLP1 E2 to continuous heparin chains in various sulfation states.

Taken together, our data support two different binding modes of APLP1 E2 to HS and HSPGs, one characterized by the specific binding to highly sulfated nonreducing ends of HS-like molecules (Fig. 6 ▶ *a*), while the other provides a general HS-binding capability (Fig. 6 ▶ *b*). These features of E2 seem to be analogous to the DNA-binding behaviour of transcription factors (TFs). In addition to very specific sequence recognition, TFs show a general DNA-binding capability. This feature enables TFs to slide along DNA strands and find specific binding sites much faster than expected for simple three-dimensional diffusion (Riggs *et al.*, 1970 ▶; Hammar *et al.*, 2012 ▶). Similarly, the soluble APP ectodomain (sAPP) could be recruited by the E2 domains to the HS chains of HSPGs by a general inherent heparin-binding capability. Concentration of sAPP at the site of action then could promote fast recruitment to nonreducing ends immediately after modification by heparanases.

We thus propose HS modification, especially HS truncation by heparanase, as a general regulatory mechanism of the HSPG–APP/APLP interaction. Such mechanisms have been demonstrated for other HSPG-binding proteins (Fux *et al.*, 2009 ▶; Christianson & Belting, 2014 ▶). Heparan sulfate modification has further been shown to influence processes such as inflammation (Li & Vlodavsky, 2009 ▶), axon outgrowth and neural regeneration (Takeuchi *et al.*, 2013 ▶). Our structural data suggest a crucial role of HSPG modification and specific HSPG recognition by the E2 domain in the physiological function of APP-family proteins.

## 5. Related literature   

The following references are cited in the Supporting Information for this article: Ashkenazy *et al.* (2010 ▶) and Celniker *et al.* (2013 ▶).

## Supplementary Material

PDB reference: apo APLP1 E2, 4rd9

PDB reference: APLP1 E2 in complex with dp12, 4rda

Supporting Information.. DOI: 10.1107/S1399004714027114/dw5120sup1.pdf

## Figures and Tables

**Table 1:** Data-collection and refinement statistics Values in parentheses are for the highest resolution shell.

Data set	Native	dp12 complex	dp12 complex, Zn peak	dp12 complex, Zn remote
Data collection				
Wavelength ()/energy (eV)	0.9184/13500	0.9184/13500	1.2827/9666	1.2861/9640
Space group	*P*2_1_2_1_2_1_	*P*3_1_21	*P*3_1_21	*P*3_1_21
Unit-cell parameters				
*a* ()	66.4	92.0	92.2	92.2
*b* ()	79.1	92.0	92.2	92.2
*c* ()	95.2	208.5	208.7	208.8
Resolution range ()	34.82.6 (2.742.60)	50.02.5 (2.652.50)	50.02.7 (2.862.70)	50.02.7 (2.862.70)
*R*_merge_ † (%)	7.2 (30.5)	6.2 (53.8)	3.9 (32.6)	4.0 (34.2)
*R*_meas_ ‡ (%)	13.9 (59.7)	7.2 (62.8)	5.1 (42.0)	5.2 (44.1)
*I*/(*I*)	17.8 (5.3)	13.5 (2.1)	15.4 (2.6)	15.3 (2.6)
Completeness (%)	100.0 (100.0)	99.3 (98.4)	96.4 (96.3)	96.4 (96.2)
Observations (total/unique)	116990/16045	137732/36051	108033/52620	108175/52654
Refinement				
*R*_work_/*R*_free_ (%)	21.5/24.7	21.6/24.5		
No. of non-H atoms				
Total	3174	3556		
Protein	3127	3295		
Heparin		192		
Other	47	69		
Solvent content (%)	56.9	77.5		
*B* factors (^2^)				
Wilson plot	57.2	61.0		
Overall	55.6	69.4		
Protein	55.7	66.5		
Heparin		121.2		
Other	49.1	58.4		
R.m.s.d., bond lengths ()	0.003	0.004		
R.m.s.d., bond angles ()	0.64	0.9		
R.m.s.d., bonded *B* factors (^2^)	4.4	4.2		
Ramachandran plot				
Favoured (%)	99.2	98.99		
Allowed (%)	0.8	1.01		
Others (%)	0	0		

† *R*_merge_ = 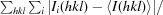 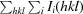.

‡ *R*_meas_ = 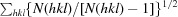 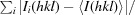 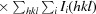.
