# Supplementary material for: Interaction of the amyloid precursor protein-like protein 1 (APLP1) E2 domain with heparan sulfate involves two distinct binding modes
Source: Acta Crystallogr D Biol Crystallogr. 2015 Feb 26;71(Pt 3):494–504. doi: 10.1107/S1399004714027114 (PMC4356362; doi:10.1107/S1399004714027114)
Supplement: Supplementary file 1 [file d-71-00494-sup1.pdf]

BIOLOGICAL
CRYSTALLOGRAPHY

Volume 71 (2015)

Supporting information for article:

Interaction of the amyloid precursor protein like protein 1 (APLP1) E2 domain with heparan sulfate involves two distinct binding modes

Sven O. Dahms, Magnus C. Mayer, Dirk Roeser, Gerd Multhaup and Manuel E. Than

S1. Materials and methods

For surface conservation analysis conservation scores were calculated with the ConSurf server (Ashkenazy *et al.*, 2010). The herein described heparin complexed APLP1-E2 structure and a pre-calculated multiple sequence alignment of the E2 domains of APP family proteins from the ConSurf-DB (Celniker *et al.*, 2013) was used as input information for the ConSurf server.

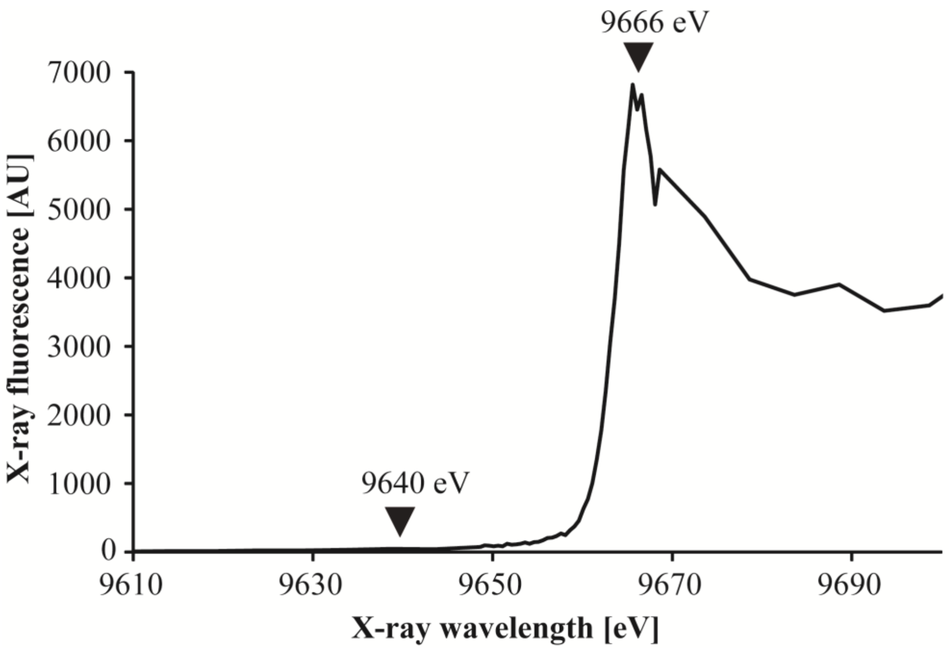

Figure S1 X-ray fluorescence scan of APLP1-E2-heparin complex crystals. The diagram indicates the fluorescence of the crystals in dependence of the X-ray energy near the zinc absorption edge. Datasets for calculation of a zinc-specific anomalous double difference density map were collected at energy values marked with arrowheads.

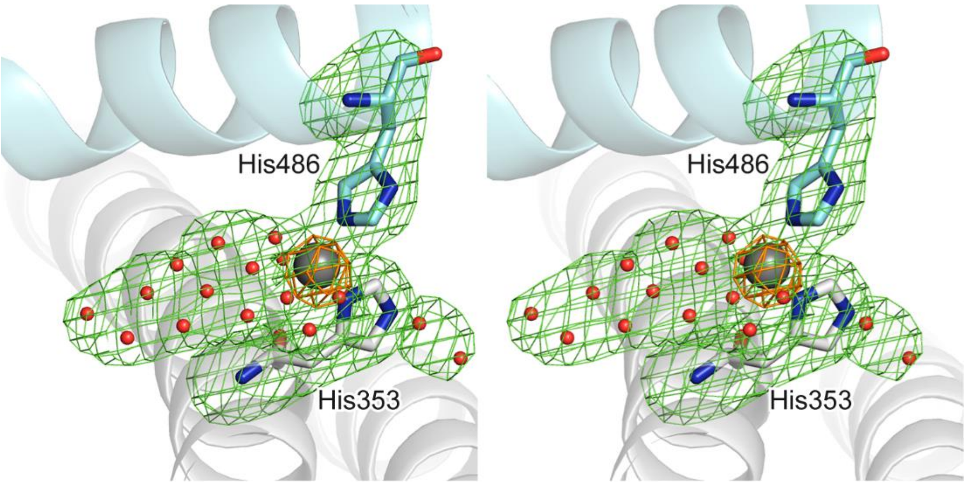

Figure S2 Crystallographic symmetry interaction mediated by zinc and an unknown zinc chelator. The stereo representation shows symmetry related APLP1-E2 protomers given as transparent cartoon representation and colored in cyan or in grey. Amino acids of symmetry related APLP1-E2 protomers involved in zinc (black sphere) coordination are shown as stick model and carbon atoms are colored in cyan or in grey. Single oxygen atoms (red spheres) were fitted into the electron density of an unknown zinc chelator during model building and structure refinement. The fo-fc kicked omit electron density map (oxygen atoms representing the unknown zinc chelator and the coordinating histidine residues were omitted) is contoured at 3.0 σ (green mesh). The zinc-specific anomalous double difference density map is contoured at 20.0 σ (orange mesh).

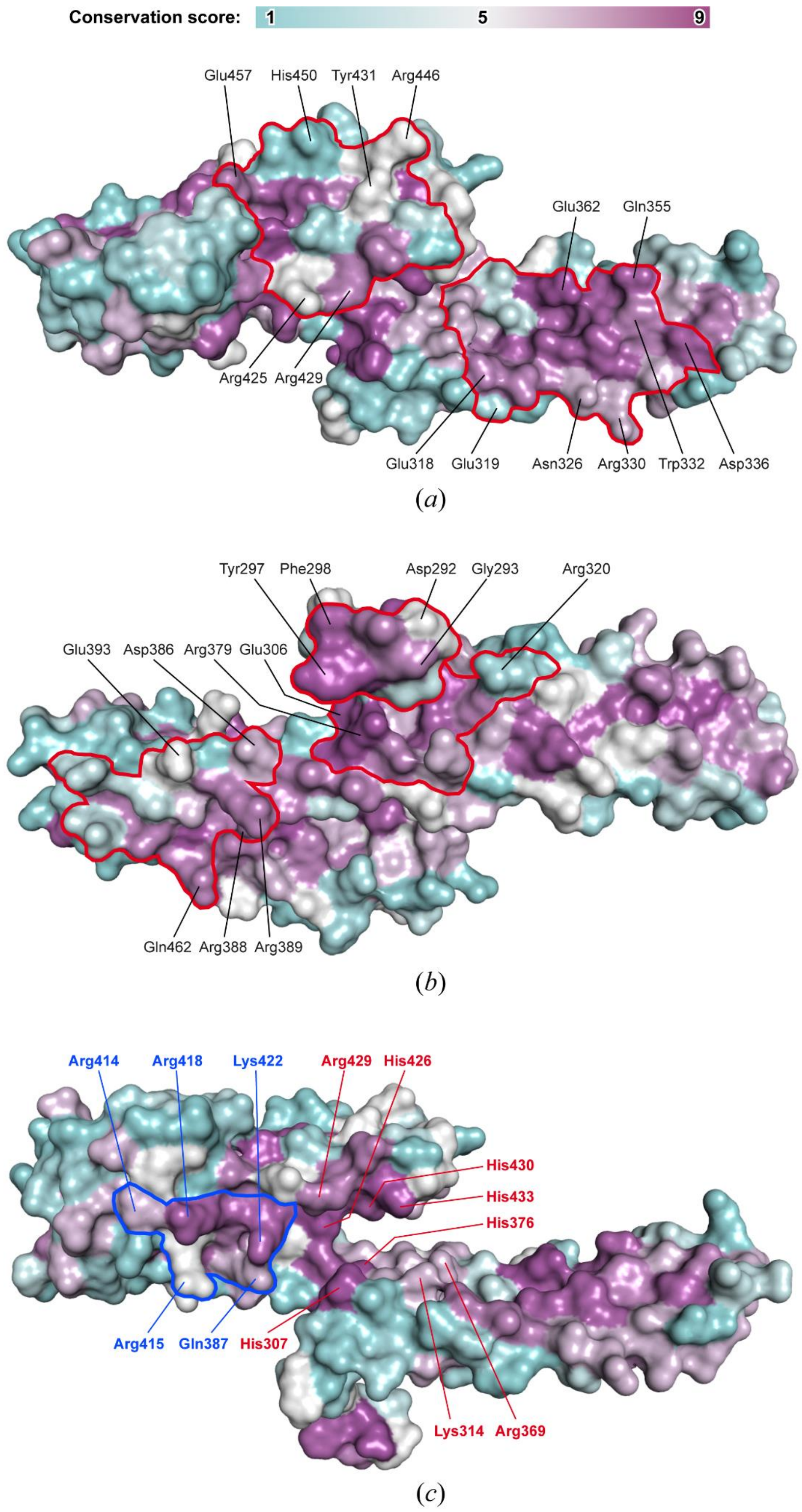

Figure S3 Analysis of the evolutionary conservation of amino acid residues located at the molecular surface of APLP1-E2. The surface representation is colored according to the conservation score, ranging from 1 (low sequence conservation) to 9 (high sequence conservation). Surface regions involved in interactions are surrounded by a red line. Selected amino acid residues involved in interactions are numbered. (a) Interaction surfaces involved in the formation of the antiparallel APLP1-E2 assembly. (b) Interaction surfaces involved in the formation of the parallel APLP1-E2

assembly. (c) Amino acids involved in specific interactions (hydrogen bonds) between APLP1-E2 and heparin are labeled in red. The positively charged surface covered by the sugar rings 4b-6b of heparin chain b is surrounded in blue (amino acids are labeled in blue).

References

Ashkenazy, H., Erez, E., Martz, E., Pupko, T. & Ben-Tal, N. (2010). *Nucleic Acids Res.* **38**, W529–W533.

Celniker, G., Nimrod, G., Ashkenazy, H., Glaser, F., Martz, E., Mayrose, I., Pupko, T. & Ben-Tal, N. (2013). *Isr. J. Chem.* **53**, 199–206.
